# Thanks to the Editorial Board of Dementia & Neuropsychology

**DOI:** 10.1590/1980-5764-DN-2023-A001

**Published:** 2023-05-05

The Editors-in-Chief of Dementia & Neuropsychology, Ricardo Nitrini and Sonia Maria Dozzi Brucki, thank the Co-Editors (2), Associate Editors (50), Advisory Board (52), Social Media and Visual Abstract Editors (2), and Reviewers (142) who dedicated their time to the editorial tasks of this journal from January 1, 2022 to December 31, 2022.

Thank you very much!

## CO-EDITORS

Eliane Correa Miotto
Mônica Sanches Yassuda

## ASSOCIATE EDITORS

Adalberto Studart-Neto
Analuiza Camozzatto de Pádua
Antonio Lucio Teixeira Junior
Artur Martins Novaes Coutinho
Breno Satler de Oliveira Diniz
Ceres Eloah de Lucena Ferretti
Claudia Berlim de Mello
Claudia Kimie Suemoto
Cláudia Maia Memória
Cleusa Pinheiro Ferri
Déborah Oliveira
Douglas Mendes Nunes
Eduardo Sturzeneker Trés
Eliasz Engelhardt
Elisa de Paula França Resende
Ezequiel Surace
Florindo Stella
Jacy Bezerra Parmera
Jerusa Smid
Joaquim Brasil Neto
Juliana Nery de Souza-Talarico
Karolina Gouveia César-Freitas
Lea Tenenholz Grinberg
Leandro Tavares Lucato
Leonardo Cruz de Souza
Leonel Tadao Takada
Lilian Schafirovits Morillo
Lucas Porcello Schilling
Maíra Okada de Oliveira
Maira Tonidandel Barbosa
Marcela Lima Silagi de Siqueira
Marcia Cristina Nascimento Dourado
Marcia Maria Pires Camargo Novelli
Marcio Luiz Figueredo Balthazar
Maria Joana Mader Joaquim
Maria Niures Pimentel dos Santos Matioli
Mario Amore Cecchini
Mauricio Arcos-Burgos
Mirna Li Hosogi
Nilton Santos Custodio Capuñay
Pablo Miguel Bagnati
Renata Kochhann
Roberta Diehl-Rodriguez
Rubens Gisbert Cury
Thaís Bento Lima da Silva
Valéria Santoro Bahia
Valeska Marinho Rodrigues
Vitor Geraldi Haase
Vitor Tumas
Wyllians Jose Vendramini Borelli

## CONSULTIVE BOARD

Alexandre Castro-Caldas
André Luis Fernandes Palmini
Andrea Camaz Deslandes
Andrew J. Lees
Benito Pereira Damasceno
Cláudia da Costa Leite
Claudia Sellitto Porto
Dora Selma Fix Ventura
Facundo F. Manes
Francisco de Assis Carvalho do Vale
Francisco Javier Lopera Restrepo
Giacomo Rizzolatti
Helenice Charchat-Fichman
Henrique Cerqueira Guimarães
Howard Chertkow
Jamary Oliveira Filho
Jerson Laks
Jerusa Smid
João Carlos Barbosa Machado
John R. Hodges
John C. Morris
Jorge Neval Moll Neto
José Luiz Sá Cavalcanti
Kenichi Meguro
Leila Maria Cardao Chimelli
Leonardo Ferreira Caixeta
Leonel Tadao Takada
Márcia Lorena Fagundes Chaves
Marcia Radanovic
Maria Alice de Mattos Pimenta Parente
Maria Rita dos Santos e Passos Bueno
Mayana Zatz
Michael D. Geschwind
M.-Marsel Mesulam
Moyses de Paula Rodrigues Chaves
Orestes Vicente Forlenza
Patricio Fuentes
Paulo Caramelli
Paulo Henrique Ferreira Bertolucci
Paulo Roberto de Brito Marques
Renato Anghinah
Ricardo F. Allegri
Ricardo de Oliveira Souza
Sandra Weintraub
Sérgio Luís Blay
Sergio Teixeira Ferreira
Tânia Marcourakis
Tales Alexandre Aversi-Ferreira
Thomas H. Bak
Vilma Regina Martins
Wilson Jacob Filho
Yves Joanette

## SOCIAL MEDIA AND VISUAL ABSTRACT EDITORS

Raphael Machado de Castilhos
Ricardo Pires Alvim

## REVIEWERS

Ariana Almirall-Sanchez
Adalberto Studart-Neto
Aline Cristina Gratão
Allan Gustavo Bregola
Alvaro Pentagna
Amanda Leopoldino Oliveira
Amer Hamdan
Analuiza Camozatto de Padua
André Palmini
Andrea C. Deslandes
Anita Neri
Aya Ashour
Bárbara Malcorra
Benito Damasceno
Breno Barbosa
Breno Cruz
Bruna Luchesi
Carlos Merege Filho
Carolina Latorraca
Ceres Ferretti
Claudia Memória
Claudia Porto
Conrado Borges
Cristiano Miosso
Daniela Farias-Itao
Einstein Camargos
Eliasz Engelhardt
Elisa de Paula Resende
Elodie Bertrand
Elpis Pavlidou
Emilio Herrera Junior
Eva Bettine de Almeida
Faheem Arshad
Fernanda Carvalho
Fernando Freua
Gabriela Cipolli
Gabriela dos Santos
Gustavo Christofoletti
Hélio Afonso Ghizoni Teive
Henrique Silva
Hyun Joo Choi
Isabela Paraguay
Isabela Thaís Machado de Jesus
Jacy Parmera
Janaine Cunha Polese
Jaqueline Rodrigues
Jerusa Smid
Jorge Libre
José Luiz Pedroso
José Parodi
José Siqueira Neto
Juan Carlos Carbajal Silva
Juliana Lira
Karen Socher
Karin Zazo Ortiz
Karolina Cesar Freitas
Karoline Carmona
Kenia Campanholo
Lais Lopes Delfino
Laiss Bertola
Laura Gonnerman
Laurent Cleret de Langavant
Leandro Valiengo
Lecio Figueira Pinto
Leonardo Caixeta
Leonardo de Souza
Leonel Takada
Liliane Além-Mar e Silva
Lizardo Cruzado
Lucas Mourão
Lucas Schilling
Luciana Cassimiro
Luciana Mascarenhas Fonseca
Luciana Ramalho Pimentel da Silva
Luciano Mariano
Luiz Kobutti Ferreira
Maila Neves
Maira Okada de Oliveira
Marcela Mansur-Alves
Marcela Silagi de Siqueira
Marcelo Brito
Marcelo Mendonca
Marcelo Valente
Márcia Radanovic
Marcio Luiz Balthazar
Marco Malaga
Marcos Castello de Oliveira
Marcus Kiiti Borges
Maria Alice Baptista
Maria Elisa Manso
Maria Isabel Freitas
Maria Luiza Giraldes de Manreza
Maria Niures Matioli
Maria Paula Foss
Maria Sheila Rocha
Maria Stefania de Simone
Maria Teresa Carthery-Goulart
Mariana Voos
Mario Amore Cecchini
Mateus Nogueira
Mateus Simabukuro
Miguel Garay
Mirna Portuguez
Monica Yassuda
Murilo Zibetti
Nariana Sousa
Nelson Torro-Alves
Neti Juniarti
Nilton Custodio Capuñay
Norberto Frota
Paula Camila Matos
Paulo Bertolucci
Pedro Barbosa
Pedro Brandão
Pedro Kowacs
Rafael Carra
Raphael Castilhos
Raphael Spera
Raquel Santos
Renan Domingues
Renata Avila
Renata Eloah de Lucena Ferretti-Rebustini
Renata Kochhann
Rodrigo dos Santos
Rodrigo Schultz
Ruth Mello
Sareh Panjeh
Simone Barreto
Stefanie Pina-Escudero
Sujata Das
Suparerk Janjarasjitt
Tamine Capato
Tania Maria Novaretti
Tatiana Belfort
Thais Bento da Silva
Thais Machado
Thiago Guimaraes
Thiago Vale
Tomas Leon
Victor Calil
Vitor Tumas
Ylmar Correa

